# Characteristics of Mucormycosis in Hematological Patients and a Death Prediction Model

**DOI:** 10.3389/fmicb.2021.784974

**Published:** 2021-12-15

**Authors:** Xiaoxu Ma, Ang Li, Weijie Cao, Huiling Li, Suping Zhang, Li Li, Haizhou Xing, Wenliang Tian, Pengfei Jiao, Jiajun Chen, Qingxian Zhang, Aiguo Xu, Lihua Xing

**Affiliations:** ^1^Department of Respiration, The First Affiliated Hospital of Zhengzhou University, Zhengzhou, China; ^2^Gene Hospital of Henan Province, Precision Medicine Center, The First Affiliated Hospital of Zhengzhou University, Zhengzhou, China; ^3^Department of Hematology, The First Affiliated Hospital of Zhengzhou University, Zhengzhou, China; ^4^College of Public Health, Zhengzhou University, Zhengzhou, China

**Keywords:** mucormycosis, hematological diseases, death prediction model, high-risk factors, random forest

## Abstract

Mucormycosis is an angioinvasive fungal infection, associated with high mortality. The aim of our study was to explore the high-risk factors and predict the death of hematological disease complicated with mucormycosis. We retrospectively analyzed clinical data of 31 patients with hematological disease complicated with mucormycosis, adopted random forest to establish the death prediction model, and validated the model in another 15 patients. The median age of the 31 cases was 46 (28–51) years, male to female ratio 1.38:1, and 90-day mortality rate 54.8%. The most common underlying disease was acute myeloid leukemia (58.1%). The main clinical symptoms were fever (100%), cough (87.1%), sputum (80.6%), chest pain (61.3%), and hemoptysis (19.4%). Reversed halo sign (83.9%) was the most common computed tomography sign. A total of 48.4% of patients also had *aspergillus* or bacterial infections. Discriminative models were constructed by random forest with 17 non-survivors and 14 survivors. Procalcitonin, the duration of intravenous administration of amphotericin B or amphotericin B liposomes, and neutropenia at death or 90 days of survival were the leading risk factors for poor prognosis, with area under the curve of 0.975 (95% CI 0.934–1). We chose 0.6775 as death prediction threshold (with 82.3% sensitivity and 100% specificity) and validated the model successfully in another 15 patients. Chest pain and reversed halo sign are specific clinical and image signs of hematological disease complicated with mucormycosis. Neutropenia, elevated procalcitonin, and insufficient use time of amphotericin B or amphotericin B liposomes are risk factors for death.

## Introduction

Mucormycosis is a rare but life-threatening fungal infection. It often occurs in patients with diabetes mellitus, hematological malignancy, solid organ transplantation, bone marrow transplantation, and trauma (Roden et al., 2005; Feng and Sun, 2018). Hematological malignancies and hematopoietic stem cell transplantation have surpassed diabetes and become the most underlying diseases of mucormycosis in developed countries (Lanternier et al., 2012; Prakash and Chakrabarti, 2019). The clinical signs and symptoms of invasive mucormycosis are non-specific, so it is difficult to discriminate between invasive mucormycosis and aspergillosis infection and the diagnosis is often delayed (Klimko et al., 2019).

Although the mortality of mucormycosis has decreased over time, it is still as high as 49.8% (Muthu et al., 2021). The mortality of hematological disease complicated with mucormycosis (HDM) is much higher than other underlying diseases up to 88% (Kennedy et al., 2016; Jestin et al., 2021), which has become an important factor leading to the death of patients with hematological diseases. More and more studies have focused on the high-risk factors of death. Studies have shown that early diagnosis and timely initiation of medical therapy are the key factors in reducing the mortality of mucormycosis (Chamilos et al., 2008; Jeong et al., 2015). Mucormycosis localization, invasive mechanical ventilation, and neutropenia are also high-risk factors for death (Lanternier et al., 2012; Jestin et al., 2021). A lymphocyte count < 100/mm^3^ at the time of diagnosis and high level of lactate dehydrogenase and APACHE II score were independent predictors for 28-day mortality (Lewis et al., 2014). A number of studies have confirmed that surgical treatment can improve survival (Skiada et al., 2011; Jeong et al., 2019a; Muthu et al., 2021). However, patients with pulmonary mucormycosis (PM), despite having localized disease, may be inoperable for several reasons such as refractory hematological disease and poor general condition (Choi et al., 2019; Muthu et al., 2021). Because there is still no effective method to reduce the high mortality of HDM, many studies are still trying to find the prognostic factors, to find the method to reduce the mortality. Because of the high mortality associated with PM, early identification of the disease is critical for an improved likelihood of survival (Agrawal et al., 2020). However, there are no relevant studies on the application of models to predict death of HDM. Establishing a predictive model can calculate the probability of over-all mortality due to mucormycosis, identify high-risk patients as early as possible, and give support to reduce mortality as soon as possible.

In this study, we collected the clinical data of 46 patients with HDM and compared the clinical characteristics, laboratory test, imaging between survivors, and non-survivors. We established a death prediction model using the clinical data of the survivors and non-survivors.

## Patients and Methods

### Study Design

This is a retrospective single-center observational study. All hematological patients diagnosed with mucormycosis are from a Chinese tertiary hospital—The First Affiliated Hospital of Zhengzhou University. Thirty-three patients between January 1, 2018, and October 31, 2020, and 16 patients between November 1, 2020, and January 31, 2021, were included in this analysis. This study was approved by the Ethics Committee of The First Affiliated Hospital of Zhengzhou University (2021-KY-0286).

### Data Collection

**FIGURE 1:**
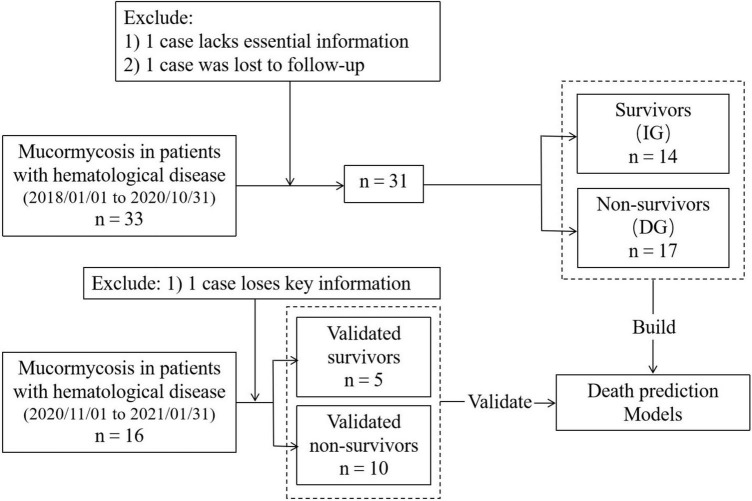
Flow diagram of included patients.

According to the information and test results provided by the electronic medical record, the patient’s demographic information, underlying diseases, clinical symptoms, laboratory examination, chest computed tomography (CT), bronchoscopy results, combined infections, and timing and agent of antifungal therapy were collected. All patients were followed up for more than 90 days. According to the prognosis of patients, they were divided into survivors and non-survivors. The survivors were called improved group (IG), and the non-survivors were called death group (DG). Regarding the controversial or missing information in the electronic medical record, we contacted the attending doctor and family members of the patient to supplement it. All data had been reviewed by a hematologist and two respiratory experts. If there was any objection, then the research team would discuss and decide. Among the 33 patients before October 31, 2020, one patient was lost to follow-up and one patient had insufficient data. Therefore, 31 cases were finally included in the study to analyze and establish a death prediction model. After November 1, 2020, missing key information of one patient was excluded. The data of 15 patients after November 1, 2020, were collected to verify the predictive effectiveness of the model. The flow diagram of included patients is shown in Figure 1.

### Definitions

The included cases were classified into proven, probable, or possible diagnosis according to the 2019 EORTC/MSG diagnostic criteria (Cornely et al., 2019).

Neutropenia: Neutrophil count <500/μl (Kara et al., 2009).

Pulmonary mucormycosis: Mucormycosis are confined to the lungs (Tedder et al., 1994).

Disseminated mucormycosis: Mucormycosis in one organ can spread hematogenously to other organs (Petrikkos et al., 2012).

Reversed halo sign (RHS): A focal groundglass attenuation surrounded by a ring of consolidation in cryptogenic organizing pneumonia on CT images (Legouge et al., 2014).

### Statistical Analysis

R 3.6.1 version for statistical analysis of cohort characteristics was adopted. Categorical variables were represented by counts and percentages [n (%)], continuous variables by median (interquartile range, IQR), and differences between groups were analyzed using Fisher’s exact test or Student’s *t*-test. One-sided *P* value < 0.05 was considered statistically significant. The random forest with leave-one-out cross-validation was used to construct the discriminant model, and the embedded reverse selection method was used for feature selection. The median selected in the relative cohort was used for model construction and verification. R program package pROC was used to visualize receiver operating characteristic (ROC) curve.

## Results

### Demographics and Clinical Characteristics

The demographics and clinical characteristics are shown in Table 1. The median age of 31 patients was 46 years (IQR 28–51), and the male-to-female ratio was 1.38:1. The median age of 14 patients in the IG was 40 years (IQR 22.8–49.8), and the median age of 17 patients in the DG 46 years (IQR 29–53), a difference not statistically significant. The total 90-day mortality of 31 patients was 54.8%, and the mortality of male was higher than that of female (66.7 vs. 38.5%), a difference not statistically significant.

**TABLE 1:** Characteristics of patients with hematological disease complicated with mucormycosis.

Characteristics	Total, *n* (%), (*n* = 31)	Model patients		*P* value	Validated patients (*n* = 15)	
		Improved group *n* (%), (*n* = 14)	Death group *n* (%), (*n* = 17)		Validated survivors *n* (%), (*n* = 5)	Validated non-survivors *n* (%), (*n* = 10)
Median age (range), years	46 (28–51)	40 (22.8–49.8)	46 (29–53)	0.413	30 (12–48)	46.5 (39.5–54.3)
Male	18 (58.1)	6 (42.9)	12 (70.6)	0.157	4 (80)	7 (70)
Underlying conditions				0.375		
AML	18 (58.1)	7 (50)	11 (64.7)		3 (60)	4 (40)
M1	1	1	0		1	0
M2	7	3	4		1	0
M3	2	1	1		0	0
M5	2	0	2		1	2
ALL	4 (12.9)	3 (21.4)	1 (5.9)		2 (40)	0 (0)
T-ALL	2	2	0		1	0
B-ALL	1	0	1		1	0
MDS	3 (9.7)	1 (7.1)	2 (11.8)		0 (0)	2 (20)
Allo-HSCT	3 (9.7)	2 (14.3)	1 (5.9)		0 (0)	2 (20)
AL	1 (3.2)	1 (7.1)	0 (0)		0 (0)	0 (0)
Aplastic anemia	1 (3.2)	0 (0)	1 (5.9)		0 (0)	1 (10)
Lymphoma	1 (3.2)	0 (0)	1 (5.9)		0 (0)	0 (0)
CMML	0 (0)	0 (0)	0 (0)		0 (0)	1 (10)
Combined diabetes	2 (6.5)	2 (14.3)	0 (0)	0.196	0 (0)	1 (10)
**Clinical features**						
Fever	31 (100)	14 (100)	17 (100)	NA	5 (100)	10 (100)
Cough	27 (87.1)	13 (92.9)	14 (82.4)	0.607	4 (80)	7 (70)
Sputum	25 (80.6)	13 (92.9)	12 (70.6)	0.185	4 (80)	5 (50)
Chest pain	19 (61.3)	9 (64.3)	10 (58.8)	1.000	4 (80)	6 (60)
Neutropenia at diagnosis	27 (87.1)	13 (92.9)	14 (82.4)	0.607	5 (100)	7 (70)
Neutropenia at death or 90 days after onset	12 (38.7)	0 (0)	12 (70.6)	<0.001	0 (0)	7 (70)
Hemoptysis	6 (19.4)	0 (0)	6 (35.3)	0.021	1 (20)	1 (10)
**Clinical forms of mucormycosis**						
Pulmonary	24 (77.4)	12 (85.7)	12 (70.6)	NA	5 (100)	9 (90)
Disseminated	7 (22.6)	2 (14.3)	5 (29.4)	NA	0 (0)	1 (10)
Lung+brain	4	0	4		0	1
Lung+spleen	2	2	0		0	0
Lung+brain+spleen	1	0	1		0	0
Involving the brain	5 (16.1)	0 (0)	5 (29.4)	0.048	0 (0)	1 (10)
Prognosis				NA		
Survival	14 (45.2)					
Non-survival	17 (54.8)					

*AML, acute myeloid leukemia; Allo-HSCT, allogeneic hematopoietic stem cell transplantation; ALL, acute lymphocytic leukemia; T-ALL, acute T lymphocytic leukemia; B-ALL, acute B lymphocytic leukemia; MDS, myelodysplastic syndrome; AL, acute leukemia; CMML, chronic myelomonocytic leukemia.*

The underlying diseases of the 31 patients were all hematological diseases, including 18 cases of acute myeloid leukemia, four acute lymphoblastic leukemia, three myelodysplastic syndrome, one aplastic anemia, one lymphoma, one acute leukemia, and three received allogeneic hematopoietic stem cell transplantation. Two patients had type 2 diabetes. There were 24 cases of PM and seven disseminated mucormycosis. Twenty-seven patients (87.1%) had neutropenia at the onset of mucormycosis. All 31 cases had fever, among which 27 (87.1%) had cough, 25 (80.6%) sputum, and 19 (61.3%) chest pain. There was no significant difference in symptoms between the IG and the DG. Six cases (19.4%) of hemoptysis died (P = 0.021).

### Imaging and Laboratory Examination

The imaging findings are shown in Table 2. All 31 cases had pulmonary involvement; meanwhile, seven cases had extrapulmonary dissemination, including four with the brain, two with the spleen, and one with the brain and the spleen. All five patients with brain involvement died (P = 0.048). The main chest CT findings were: 26 cases (83.9%) with RHS, 24 (77.4%) with cavities, nine (29.0%) with pleural effusion, seven (22.6%) with nodules, and three (9.7%) with consolidation. There was no obvious regularity in the distribution of mucor lesions in the lungs, but the patients in the IG mostly had single lesions (78.6%), and the patients in the DG had a higher proportion of multiple lesions (58.8%).

**TABLE 2:** Manifestations on chest CT in patients with pulmonary mucormycosis.

Manifestations on chest CT	Total, *n* (%), (*n* = 31)	Model patients		*P* value	Validated patients (*n* = 15)	
		Improved group *n* (%), (*n* = 14)	Death group *n* (%), (*n* = 17)		Validated survivors *n* (%), (*n* = 5)	Validated non-survivors *n* (%), (*n* = 10)
**Pulmonary features**						
Reversed halo sign	26 (83.9)	10 (71.4)	16 (94.1)	0.148	5 (100)	9 (90)
Consolidation	3 (9.7)	2 (14.3)	1 (5.9)	0.576	0 (0)	0 (0)
Nodule	7 (22.6)	2 (14.3)	5 (29.4)	0.412	0 (0)	3 (30)
Pleural effusion	9 (29.0)	4 (28.6)	5 (29.4)	1.000	1 (20)	4 (40)
Cavity	24 (77.4)	13 (92.9)	11 (64.7)	0.094	1 (20)	0 (0)
**Distribution**						
Unilateral upper lobe	10 (32.3)	7 (50)	3 (17.6)	0.121	2 (40)	2 (20)
Right	6 (19.4)	3	3		1	0
Left	4 (12.9)	4	0		1	2
Unilateral lower lobe	7 (22.6)	3 (21.4)	4 (23.5)	1.000	0 (0)	1 (10)
Right	3 (9.7)	1	2		0	0
Left	4 (12.9)	2	2		0	1
Middle lobe	1 (3.2)	1 (7.1)	0 (0)	NA	1 (20)	2 (20)
Unilateral multilobular lesions	3 (9.7)	1 (7.1)	2 (11.8)	NA	0 (0)	2 (20)
Right	1 (3.2)	0	1		0	2
Left	2 (6.5)	1	1		0	0
Bilateral lesions	10 (32.3)	2 (14.3)	8 (47.1)	0.068	2 (40)	3 (30)
Lesions				0.067		
Single	18 (58.1)	11 (78.6)	7 (41.2)		3 (60)	5 (50)
Multiple	13 (41.9)	3 (21.4)	10 (58.8)		2 (40)	5 (50)

Laboratory examination results are shown in Table 3. The median white blood cell count at the onset of mucormycosis of 31 patients was 0.5 × 10^9^/L (IQR 0.35–0.9), the median neutrophil count 0.09 × 10^9^/L (IQR 0.02–0.34), and the median platelet count 22 × 10^9^/L (IQR 11–40). C-reactive protein was elevated in all patients. The median procalcitonin (PCT) [0.31 ng/ml (IQR 0.191–1.48)] in the DG was higher than that in the IG [0.1225 ng/ml (IQR 0.1075–0.28)], but the difference was not statistically significant. Among the 31 patients with mucormycosis, 15 were proven, nine probable, and seven possible. Fourteen cases were proven by lung tissue pathology and one by culture of pleural effusion; seven cases were positive for mucor by metagenomic next-generation sequencing; mucor hyphae were detected in four cases by immunofluorescence of bronchoalveolar lavage fluid (Balf); one case was positive by sputum culture, and one case by Balf culture. Eleven (64.7%) cases in the DG and four (28.6%) cases in the IG had bacterial or other fungal infections, a difference not statistically significant.

**TABLE 3:** Results of laboratory examination, diagnosis, and treatment.

Results	Total, *n* (%), (*n* = 31)	Model patients		*P* value	Validated patients (*n* = 15)	
		Improved group *n* (%), (*n* = 14)	Death group *n* (%), (*n* = 17)		Validated survivors *n* (%), (*n* = 5)	Validated non-survivors *n* (%), (*n* = 10)
Laboratory examination						
WBC (10^9/L)	0.5 (0.35–0.9)	0.38 (0.2–0.75)	0.8 (0.43–0.9)	0.079	0.33 (0.27–0.73)	0.475 (0.2925–1.7675)
NE (10^9/L)	0.09 (0.02–0.34)	0.04 (0.01–0.105)	0.17 (0.05–0.43)	0.094	0.02 (0.01–0.05)	0.17 (0.0325–0.5375)
Lymphocyte (10^9/L)	0.23 (0.095–0.505)	0.32 (0.088–0.508)	0.17 (0.1–0.41)	0.526	0.31 (0.26–0.51)	0.155 (0.14–0.31)
Monocyte (10^9/L)	0.02 (0.005–0.085)	0.01 (0.0025–0.02)	0.03 (0.01–0.25)	0.104	0 (0–0.01)	0.015 (0–0.315)
Platelet (10^9/L)	22 (11–40)	24.5 (14.75–39.5)	22 (7–39)	0.257	22 (6–24)	22 (14.25–30)
Albumin (g/L)	31.1 (25.65–34.85)	32.1 (29.8–36.925)	26.6 (24.3–33.2)	0.060	32.2 (31.6–32.7)	30.75 (27.675–32.375)
LDH (U/L)	359 (259.25–586.75)	285 (171.5–618.5)	368 (297–426)	0.299	242 (68–263)	258 (202–485)
CRP (mg/L)	123.57 (93.44–206.38)	118.21 (83.71–137.04)	123.57 (101.1–220.8)	0.468	68.23 (51.15–149.2)	108.4 (71.26–146.57)
PCT (ng/ml)	0.22 (0.1225–0.7925)	0.1225 (0.1075–0.28)	0.31 (0.191–1.48)	0.588	0.65 (0.242–0.74)	0.465 (0.198–0.926)
G test	0 (0)	0 (0)	0 (0)	NA	0 (0)	0 (0)
GM test	0 (0)	0 (0)	0 (0)	NA	1 (20)	2 (20)
Methods of diagnosis				NA		
Histopathology	14 (45.2)	11 (78.6)	3 (17.6)		0 (0)	0 (0)
Immunofluorescence	4 (12.9)	2 (14.3)	2 (11.8)		2 (40)	1 (10)
mNGS	7 (22.6)	0 (0)	7 (41.2)		5 (100)	9 (90)
Culture	3 (9.7)	0 (0)	3 (17.6)		0 (0)	0 (0)
Pleural effusion	1	0	1		0	0
Sputum	1	0	1		0	0
Balf	1	0	1		0	0
Concurrent infection	15 (48.4)	4 (28.6)	11 (64.7)	0.156	3 (60)	5 (50)
Combined with *Aspergillus*	1 (3.2)	0 (0)	1 (5.9)		1 (20)	0 (0)
Combined with *AB*	3 (9.7)	0 (0)	3 (17.6)		0 (0)	0 (0)
Combined with *KP*	3 (9.7)	1 (7.1)	2 (11.8)		0 (0)	1 (10)
Combined with *PA*	3 (9.7)	1 (7.1)	2 (11.8)		0 (0)	0 (0)
Combined with PJ	0 (0)	0 (0)	0 (0)		0 (0)	1 (10)
Combined with *EF*	1 (3.2)	0 (0)	1 (5.9)		0 (0)	1 (10)
Combined with *SM*	0 (0)	0 (0)	0 (0)		1 (20)	0 (0)
Combined with *Aspergillus+ PA*	1 (3.2)	1 (7.1)	0 (0)		0 (0)	1 (10)
Combined with *Aspergillus+ KP*	0 (0)	0 (0)	0 (0)		0 (0)	1 (10)
Combined with *AB+ KP*	1 (3.2)	0 (0)	1 (5.9)		0 (0)	0 (0)
Combined with *KP+ PA*	1 (3.2)	1 (7.1)	0 (0)		0 (0)	0 (0)
Combined with *KP+ EF*	1 (3.2)	0 (0)	1 (5.9)		0 (0)	0 (0)
Combined with *PJ + EF + SM*	0 (0)	0 (0)	0 (0)		1 (20)	0 (0)
Treatment						
Prior Voriconazole use	31 (100)	14 (100)	17 (100)	NA	5 (100)	9 (90)
Medication				NA		
PCZ	2 (6.5)	0 (0)	2 (11.8)		0 (0)	0 (0)
L-AMB + PCZ	13 (41.9)	8 (57.1)	5 (29.4)		1 (20)	0 (0)
AMB + PCZ	5 (16.1)	4 (28.6)	1 (5.9)		3 (60)	4 (40)
L-AMB +CAS	1 (3.2)	0 (0)	1 (5.9)		0 (0)	0 (0)
PCZ + CAS	3 (9.7)	0 (0)	3 (17.6)		0 (0)	1 (10)
L-AMB + PCZ + CAS	4 (12.9)	1 (7.1)	3 (17.6)		0 (0)	0 (0)
AMB + PCZ + CAS	2 (6.5)	1 (7.1)	1 (5.9)		1 (20)	4 (30)
L-AMB or AMB medication (I.V.)	25 (80.6)	14 (100)	11 (64.7)	0.021	5 (100)	8 (80)
L-AMB or AMB total doses (mg)	555 (32.5–1130)	1,130 (401.25–1229)	90 (0–710)	0.010	1,236 (680–1400)	307.5 (82.5–637.5)
L-AMB or AMB daily doses (mg)	26.75 (13.75–47.78)	40.44 (23.95–49.79)	22.5 (0–34.74)	0.018	30 (19.4–46.7)	25 (20.625–39.025)
L-AMB or AMB medication time (days)	15 (2.5–23.5)	23.5 (10–26.25)	4 (0–18)	0.012	35 (30–65)	11.5 (4–15)
L-AMB or AMB medication through bronchoscope	12 (38.7)	7 (50)	5 (29.4)	0.288	1 (20)	1 (10)
L-AMB or AMB nebulization	23 (71.2)	10 (71.4)	13 (76.5)	1.000	4 (80)	5 (50)
PCZ plasma concentration (>1.25 μg/mL)	10 (32.3)	7 (50)	3 (17.6)	0.121	5 (100)	1 (10)
Surgery	4 (12.9)	4 (28.6)	0 (0)	0.032	0 (0)	0 (0)
Time from mucormycosis onset to treatment (days)	4.5 (1–11)	4 (1–11)	5 (1–11)	0.631	13 (9–15)	1 (0–2)

*WBC, white blood cell; NE, neutrophile granulocyte; LDH, lactate dehydrogenase; CRP, C-reactive protein; PCT, procalcitonin; G test, 1, 3-β-D-glucan antigen test; GM test, galactomannan antigen test; mNGS, metagenomic next-generation sequencing; Balf, bronchoalveolar lavage fluid; AB, Acinetobacter baumannii; KP, Klebsiella pneumoniae; PA, Pseudomonas aeruginosa; PJ, Pneumocystis jeroveci; EF, Enterococcus faecium; SM, Stenotrophomonas maltophilia; PCZ, Posaconazole; CAS, Caspofungin; L-AMB, amphotericin B liposome; AMB, amphotericin B; I.V., intravenous injection.*

### Treatment Status

**FIGURE 2:**
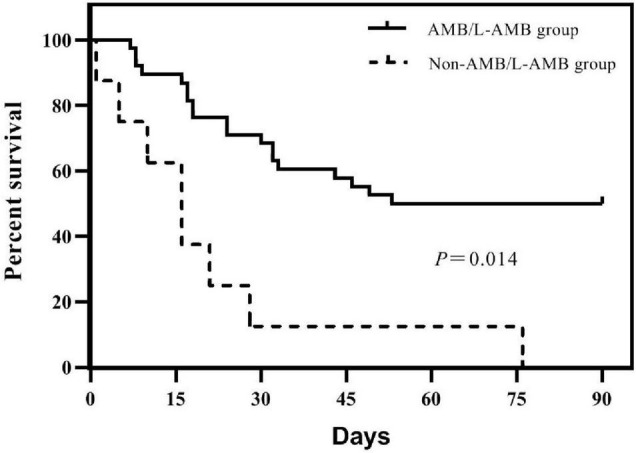
Survivorship curves of 46 patients in groups treated with amphotericin B (AMB) or amphotericin B liposome (L-AMB) or not. Survival is significantly lower in patients treated without AMB or L-AMB (*P* = 0.014).

Before the diagnosis of mucormycosis, all 31 patients had received voriconazole for antifungal prophylaxis. After diagnosis of mucormycosis, 29 patients were treated with a combined treatment plan. Thirteen cases were treated with amphotericin B liposomes (L-AMB) combined with posaconazole (PCZ), with a mortality of 38.5%; five case with amphotericin B (AMB) combined with PCZ, with mortality of 20%; four with L-AMB, PCZ, and caspofungin, with mortality of 75%; two with AMB, PCZ, and caspofungin, with mortality of 50%; and one with L-AMB combined with caspofungin, three with PCZ combined with caspofungin, and one untreated, and all these patients died. In the IG, the median cumulative dose of AMB, the median intravenous administration time, and the median daily dose of AMB or L-AMB were all significantly higher than those of the DG [1,130 mg (IQR 401.25–1,229) vs. 90 mg (IQR 0–710), *P* = 0.01; 23.5 days (IQR 10–26.25) vs. 4 days (IQR 0–18), *P* = 0.012; 40.44 mg (IQR 23.95–49.79) vs. 22.5 mg (IQR 0–34.74), *P* = 0.018]. Ninety-day mortality was significantly different between the group treated with AMB or L-AMB and the group treated without AMB or L-AMB (50 vs. 100%, *P* = 0.014) (Figure 2). Four patients who underwent surgical resection were alive (*P* = 0.032). The median time from the onset of mucormycosis to treatment of 31 patients was 4.5 days (IQR 1–11), and the difference between the IG [4 days (IQR 1–11)] and the DG [5 days (IQR 1–11)] was not statistically significant.

### Model

**FIGURE 3:**
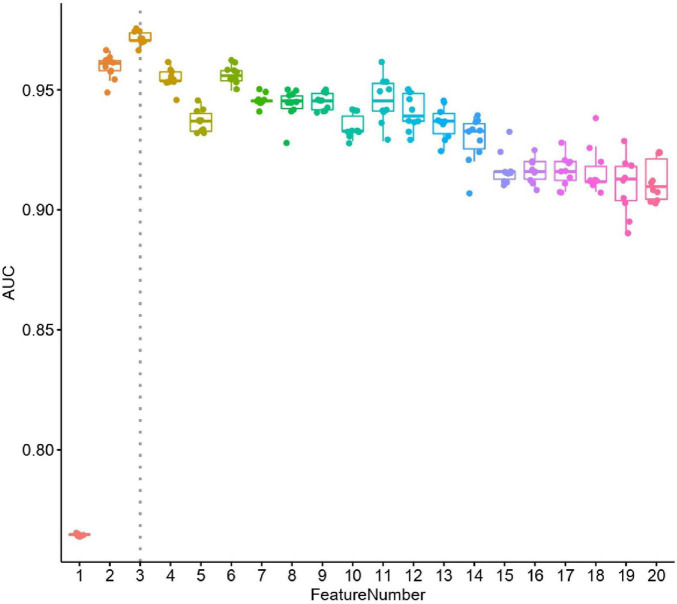
Performance of selected features. X axis is number of features used in random forest leave-one-out cross validation models, y axis is area under curve (AUC). For each random forest model, the prediction process was repeated ten times.

**FIGURE 4:**
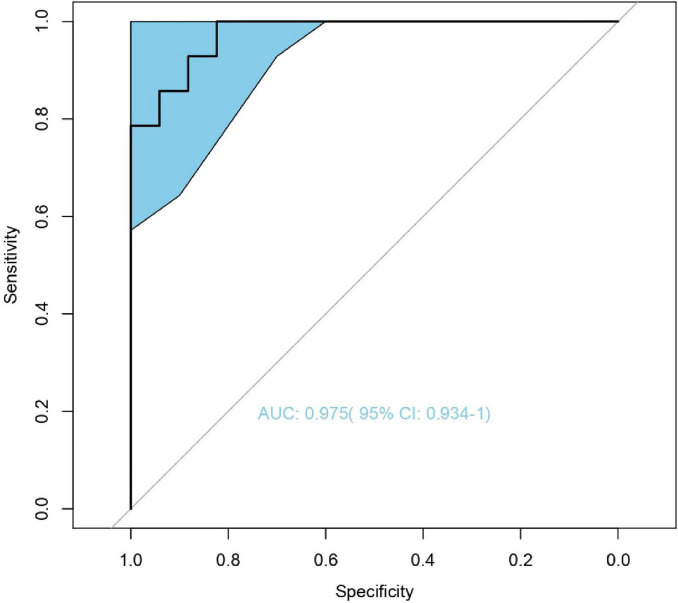
Receiver operating characteristic (ROC) curve of leave-one-out cross validation random forest models by incorporating procalcitonin, medication time and neutropenia in training cohort. (Improved group is 14, death group is 17).

**FIGURE 5:**
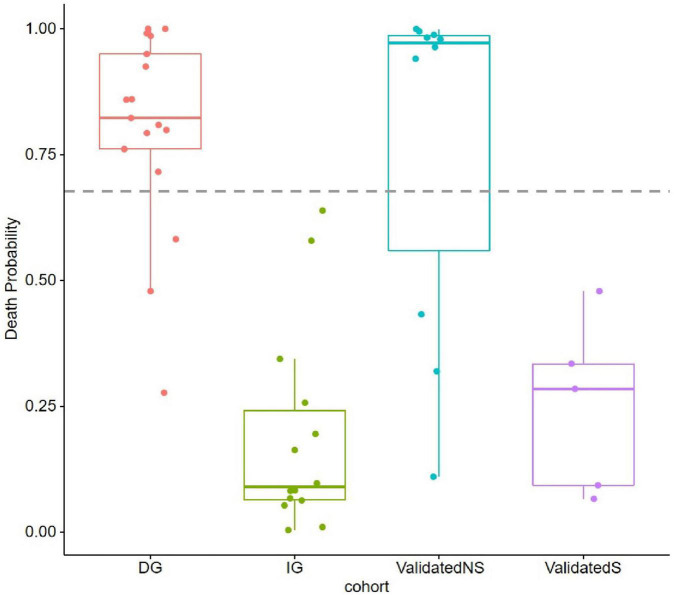
Probability of death (POD) predicted by using random forest models in training cohort and validation cohort. POD 0.6775 was set as threshold.

**FIGURE 6:**
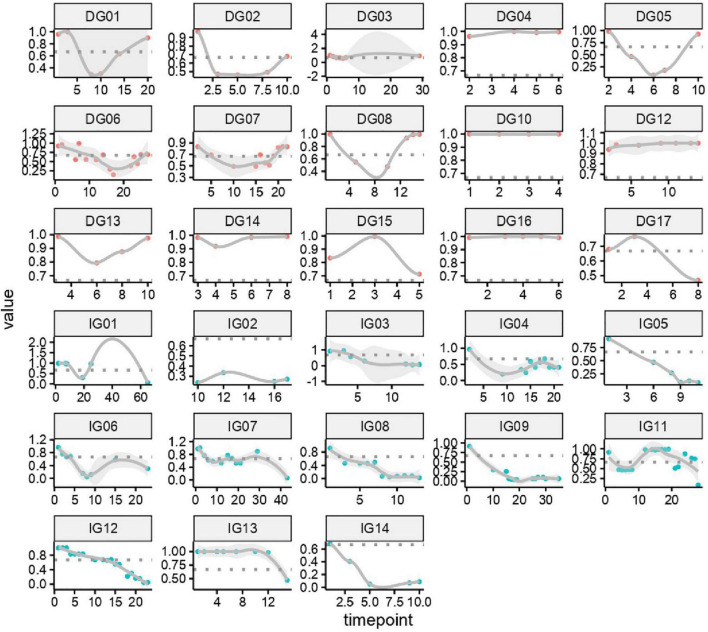
Dynamic probability of death predicted by using random forest models in training cohort. Each panel indicated one patient. X axis is time points during the whole medication process, y axis is probability of death.

We constructed a classification model to assess the death risk of patients with mucormycosis. The ROC curve was used to analyze the area under the curve (AUC) to evaluate the performance of the model. Demographic characteristics, clinical symptoms, and laboratory test results at the onset of mucormycosis were combined to construct a model. PCT, the duration of intravenous administration of AMB or L-AMB, and neutropenia at death or 90 days of survival were the most important predictors (Table 4). These three indexes were used to build the model that had the best prediction performance (Figure 3), with an AUC value of 0.975 (95% CI: 0.934–1) (Figure 4). In addition, 0.6775 was selected as the death prediction threshold, with a sensitivity of 82.3% and a specificity of 100% (Figure 4). The 10 non-survivors and five survivors from November 1, 2020, to January 31, 2021, were used as independent cohorts to verify the model. Seven of the 10 non-survivors (70%) had a death prediction value higher than 0.6775, and death prediction values of five survivors (100%) were all lower than 0.6775 (Figure 5). PCT, the duration of intravenous administration of AMB or L-AMB, and neutropenia were used to build dynamic probability of death predicted model by using random forest models (Figure 6). Each panel indicated one patient, and the dynamic death probability of the DG was generally higher than the IG.

**TABLE 4:** Importance of features in death risk prediction model.

Feature	Mean decrease Gini	Feature	Mean decrease Gini
Neutropenia (at the time of death or 90 days after onset)	1.931	Gender	0.127
Procalcitonin (PCT)	1.116	Cavity	0.117
The duration of intravenous administration of AMB or L-AMB	0.972	*Acinetobacter baumannii*	0.105
AMB/L-AMB cumulative dose	0.907	Reversed halo sign	0.094
AMB/L-AMB daily dose	0.809	*Pseudomonas aeruginosa*	0.079
Platelet	0.757	Bronchoscope medication	0.067
Time from hematological malignancy to mucormycosis	0.743	Diabetes mellitus	0.060
Lymphocyte count	0.641	*Aspergillus*	0.050
Albumin	0.637	Nodule	0.041
AMB/L-AMB nebulization time	0.606	Involve spleen	0.040
Lactic dehydrogenase	0.578	*Klebsiella pneumoniae*	0.033
Monocyte count	0.373	Chest pain	0.031
C-reactive protein	0.344	Nebulization	0.030
Neutrophil count	0.336	*Mucor*	0.030
Neutrophil percentage	0.324	*Rhizopus microsporus*	0.029
Age	0.321	Pleural effusion	0.028
White blood cell count	0.317	Cough	0.026
Lesion site	0.286	Neutropenia at diagnose	0.025
Time from mucormycosis onset to treatment	0.255	*Lichtheimia ramosa*	0.020
Hemoptysis	0.227	*Enterococcus faecium*	0.017
Surgery	0.184	Consolidation	0.016
Posaconazole plasma concentration	0.167	*Rhizomucor pusillus*	0.010
Co-infection	0.154	*Rhizopus*	0.009
Involve brain	0.144	*Rhizomucor miehei*	0.006
Sputum	0.128	*Mucor racemosus*	0.003

*AMB, amphotericin B; L-AMB, amphotericin B liposome; Mean Decrease Gini, mean decrease in Gini is the average (mean) of a variable’s total decrease in node impurity, weighted by the proportion of samples reaching that node in each individual decision tree in the random forest. A higher mean decrease in Gini indicates higher variable importance.*

## Discussion

The 31 patients in the study to establish a death prediction model and the 15 patients used for model validation were all from The First Affiliated Hospital of Zhengzhou University. Although it was a single-center study, because of the rarity of mucor infections, this is the largest single-center study to date about HDM in China. Hematological malignancy was independently associated with a worse prognosis (Claustre et al., 2020). This is the first study to use a death prediction model to predict the prognosis of patients with HDM. The model has been uploaded to the internet for clinicians to predict the probability of death of such patients, identify high-risk groups as soon as possible, and provide early intervention and treatment to improve the survival rate of patients with HDM.

All patients in our study had pulmonary involvement, but no rhino-orbital-cerebral mucormycosis was found. Jeong et al. (2019b) in their retrospective analysis showed that underlying hematological malignancy was associated with increased risk of disseminated mucormycosis and that neutropenia was significant for PM (Claustre et al., 2020), which is consistent with our study. The main clinical manifestations of PM are fever, cough, sputum, chest pain, and hemoptysis. Fever, cough, and sputum are common clinical manifestations of lung infections, which are not specific (Feng and Sun, 2018), but the sputum characteristics are different from other infections. In our study, the sputum of most patients was yellow or brown viscous sputum and was sticky and difficult to cough up, Of the 31 patients, 61.3% had chest pain, and 66.7% of the patients for model validation had chest pain, suggesting that, if patients with hematological diseases have chest pain, then the possibility of mucormycosis should be considered and chest CT should be performed as soon as possible to confirm the diagnosis. Mucormycosis is often angioinvasive and is prone to fatal hemoptysis. In this study, 19.4% of patients with massive hemoptysis died, which showed that massive hemoptysis indicated a poor prognosis.

The main signs on chest CT of 31 patients used to establish the model in this study were RHS, pulmonary consolidation, nodules, pleural effusion, and cavities, and the distribution of lesions had no obvious regularity. Previous studies have shown that the RHS is a more specific manifestation of hematological diseases with PM (Jung et al., 2015). Legouge et al. (2014) reported that, among leukemia patients with PM, a typical RHS was seen on 94% of their chest CT between day 0 and day 5 and on 64% between day 6 and day 14 and was no longer observed after day 14. In our study, 83.9% of the patients showed RHS on chest CT, which is consistent with the results of previous studies, suggesting that the RHS can be regarded as a sentinel point of hematological disease complicated with PM. Of the patients, 77.4% had irregular pulmonary cavities at different times in the course of the disease, but generally after 2 weeks of treatment, it would be difficult to distinguish between pulmonary cavities and other infections, which reminds us that, when patients with hematological diseases show respiratory symptoms, chest CT should be performed timely, so as not to miss the typical imaging signs and delay the diagnosis.

The guidelines recommend PCZ as primary prophylaxis drug in high-risk immunocompromised patients, but in our study, all 31 patients had received voriconazole for antifungal prophylaxis before the onset of mucormycosis. There is only PCZ oral suspension in our hospital, and the PCZ plasma concentration is usually insufficient, which may be lead to poor clinical effectiveness (Jia et al., 2020). Therefore, the doctors in our hospital often choose voriconazole to prevent fungal infections. The guidelines recommended liposomal amphotericin B of 5–10 mg/kg per day as first-line treatment across all patterns of organ involvement and whether the combination antifungal therapy is better than single antifungal therapy for mucormycosis is still controversial (Cornely et al., 2019), which needs to be confirmed by prospective, randomized, and double-blind clinical trials. In our study, most patients were treated with AMB or L-AMB combined with PCZ as first-line antifungal treatment for invasive mucormycosis. The substantial toxicity limited the dosage of AMB or L-AMB, and the average dose of AMB or L-AMB was less than 0.7 mg/kg, according to the guidelines which is far from enough to treat mucormycosis. Of the patients, 100% had adverse reactions such as nephrotoxicity, nausea, vomiting, and hypokalemia, which is the main reason leading to insufficient dosage. According to the research from Europe and America, the dosage of L-AMB for the treatment of mucormycosis is generally more than 3 mg/kg (Nosari et al., 2000; Moen et al., 2009; Lanternier et al., 2015), but there is a lack of L-AMB with less toxicity in China, and the dosage is generally less than 1 mg/kg. In our study, although the dosage of AMB or L-AMB was generally insufficient, the effect of dosage and usage time of AMB or L-AMB on prognosis was observed. The dosage and usage time of AMB or L-AMB in the DG were significantly less than those in the IG. Therefore, although the dosage of AMB or L-AMB is insufficient, prolonging the usage days and increasing the dosage can still improve the prognosis of patients with mucormycosis. PCZ plasma concentration in some patients was monitored, but 50% of that did not reach 1.0 mmol/L. Previous studies have shown that insufficient PCZ plasma concentration indicated a high failure rate of prophylaxis and therapy of invasive fungal disease (Chae et al., 2015; Jia et al., 2020). Insufficient PCZ plasma concentration may also be an important factor in the high mortality of patients in our study; however, in our study, only some patients had measured PCZ plasma concentration, so it has not been used to establish the death prediction model.

We used random forest to establish the death prediction model and dynamic probability of death predicted model, which showed that neutropenia, elevated PCT, and the duration of intravenous administration of AMB or L-AMB had the greatest impact on death. The death prediction model chose 0.6775 as the threshold, with sensitivity of 82.3% and specificity of 100%, and it was successfully validated in 15 cases after November 2020. Several studies show that neutropenia was associated with mortality of mucormycosis (Kara et al., 2009; Kyvernitakis et al., 2016), which is agreement with our study. It is known that the increase in PCT indicates bacterial infection. Of the 31 patients, 48.4% had other fungal or bacterial infections. Lin et al. (2017) also found that concurrent bacteremia was independently associated with 28-day mortality. In fact, the proportion of bacterial infections was much higher than 48.4% because bacterial or fungal culture has a low positive rate. In our study, the total dose, usage time, and daily dose of AMB or L-AMB used in the DG were significantly lower than those in the IG, suggesting that the use of AMB or L-AMB is an important factor to improve the prognosis. Among the total dose, usage time, and daily dose of AMB or L-AMB, mean decrease Gini of the usage time was the biggest with 0.972, suggesting that, in the case of insufficient dosage, prolonging the usage time helps improve the prognosis. Therefore, under limited conditions, actively improving the neutropenia state, controlling the combined bacterial infection and actively applying AMB or L-AMB are important measures to improve the prognosis.

This study has certain limitations. First, because of the rarity of mucormycosis, the number of cases used to establish the model is small, and the sample size needs to be expanded in the later research. Second, this is a retrospective study, although the controversial information extracted from the electronic medical record have all been verified with the family or the attending doctor of the patient, but there may still be certain deviations. Third, not all patients had measured PCZ plasma concentration, so the impact of PCZ plasma concentration on the prognosis of patients cannot be statistically analyzed. In addition, this death prediction model was established on the basis of the clinical data of patients with HDM. Whether it is applicable to patients with diabetes and other underlying diseases and mucormycosis needs further research and validation.

Our study conducted in-depth digging on the clinical data of the included patients and established the first death model of HDM, which can accurately predict the prognosis of such patients. The application of this model to the clinic can detect patients at high risk of death early, and intervention measures can be taken as soon as possible to improve the prognosis of patients.

## Data Availability Statement

The original contributions presented in the study are included in the article/supplementary material, further inquiries can be directed to the corresponding author/s.

## Ethics Statement

The studies involving human participants were reviewed and approved by the Ethics Committee of the First Affiliated Hospital of Zhengzhou University. Written informed consent from the participants’ legal guardian/next of kin was not required to participate in this study in accordance with the national legislation and the institutional requirements.

## Author Contributions

LX, AX, and QZ conceived the project. WC, HL, SZ, LL, HX, WT, PJ, and JC collected the cases and clarified the data. XM and AL analyzed and interpreted patient data. XM wrote the manuscript. AL built the model. All authors have read and approved the final manuscript.

## Conflict of Interest

The authors declare that the research was conducted in the absence of any commercial or financial relationships that could be construed as a potential conflict of interest.

## Publisher’s Note

All claims expressed in this article are solely those of the authors and do not necessarily represent those of their affiliated organizations, or those of the publisher, the editors and the reviewers. Any product that may be evaluated in this article, or claim that may be made by its manufacturer, is not guaranteed or endorsed by the publisher.

## Abbreviations

AMB: amphotericin B
AUC: area under the curve
Balf: bronchoalveolar lavage fluid
CT: computed tomography
DG: death group
HDM: hematological disease complicated with mucormycosis
IG: improved group
IQR: interquartile range
L-AMB: amphotericin B liposomes
PCT: procalcitonin
PCZ: posaconazole
PM: pulmonary mucormycosis
RHS: reversed halo sign
ROC: receiver operating characteristic.
